# Increased Expression of Integrin Alpha 6 in Nucleus Pulposus Cells in Response to High Oxygen Tension Protects against Intervertebral Disc Degeneration

**DOI:** 10.1155/2021/8632823

**Published:** 2021-10-18

**Authors:** Zeng Xu, Jiancheng Zheng, Ying Zhang, Huiqiao Wu, Bin Sun, Ke Zhang, Jianxi Wang, Fazhi Zang, Xingkai Zhang, Lei Guo, Xiaodong Wu

**Affiliations:** ^1^Department of Orthopedics, Changzheng Hospital, The Naval Medical University, Shanghai, China; ^2^Shanghai Key Laboratory for Bone and Joint Diseases, Shanghai Institute of Orthopaedics and Traumatology, Shanghai Ruijin Hospital, Shanghai Jiaotong University School of Medicine, China

## Abstract

The destruction of the low oxygen microenvironment in nucleus pulposus (NP) cells played a critical role in the pathogenesis of intervertebral disc degeneration (IVDD). The purpose of this study was to determine the potential role of integrin alpha 6 (ITG *α*6) in NP cells in response to high oxygen tension (HOT) in IVDD. Immunofluorescence staining and western blot analysis showed that the levels of ITG *α*6 expression were increased in the NP tissue from IVDD patients and the IVDD rat model with mild degeneration, which were reduced as the degree of degeneration increases in severity. In NP cells, the treatment of HOT resulted in upregulation of ITG *α*6 expression, which could be alleviated by blocking the PI3K/AKT signaling pathway. Further studies found that ITG *α*6 could protect NP cells against HOT-induced apoptosis and oxidative stress and protect NP cells from HOT-inhibited ECM protein synthesis. Upregulation of ITG *α*6 expression by HOT contributed to maintaining NP tissue homeostasis through the interaction with hypoxia-inducible factor-1*α* (HIF-1*α*). Furthermore, silencing of ITG *α*6 *in vivo* could obviously accelerate puncture-induced IVDD. Taken together, these results revealed that the increase of ITG *α*6 expression by HOT in NP cells might be a protective factor in IVD degeneration as well as restore NP cell function.

## 1. Introduction

Intervertebral disc degeneration (IVDD) is the most common musculoskeletal disorder and a major cause of disability [1–3]. The development of IVDD is directly associated with several factors, such as aging, genetic susceptibility, body weight, heavy workload, and smoking [4, 5]. The intervertebral disc (IVD) mainly consists of three tissue compartments: the outer annulus fibrosus (AF), the inner nucleus pulposus (NP), and the cephalic and caudal cartilage endplates [6]. NP and AF tissues work together to provide the major anatomic functions of the IVD: spinal flexibility and load distribution [7]. NP cells play the core role in the maintenance of IVD function [6]. The number and function of NP cells are decreased, and the secretion of inflammatory factors is increased with increasing age in humans, which is characterized by increased matrix catabolism and fibrosis accompanied by changes in the NP cell phenotype [8, 9].

IVD is the largest ischemic and hypoxic tissue in mammals due to a complete lack of tissue vascularity [10]. Thus, NP cells are characterized by low oxygen tension. Hypoxia plays an important role in maintaining the physiological functions of NP cells, such as energy metabolism, matrix synthesis, and cell viability [8, 9]. NP cells could ensure their function and survival in a low oxygen environment through the regulation of cell apoptosis, the generation of ROS [11], and some critical gene expressions, such Sox9 and Collagen II alpha 1 (Col2*α*1). Growing evidence suggested that neovascularization in discs increased oxygen tension in the progression of IVDD, which led to perturbation of the low oxygen gradient and the change of cell metabolism [12, 13]. However, the exact mechanisms by which the change of the low oxygen microenvironment initiates IVDD are not fully elucidated.

Integrins (ITG), which are well-known receptors, are important in the communication between cells and their surrounding tissues or extracellular matrix (ECM) to regulate cell apoptosis, migration, proliferation, and survival [14]. Integrins are composed of two distinct chains: alpha (*α*) subunits and beta (*β*) subunits. *α* and *β* subunits have been discovered in vertebrates, which totally assemble into 24 distinct integrin complexes [15]. Increasing evidence suggested that integrins were involved in the pathogenesis of IVDD [16–18]. As compared to the adjacent annulus fibrosus region, several integrin subunits were more highly expressed in nucleus pulposus cells, such as ITG *β*4,*α*3, *α*5, and *α*6 [19]. Recently, studies showed that ITG *α*6 was involved in the regulation of the production of ROS and cell apoptosis by hypoxia in some organs and tissues [20, 21]. However, it is unclear whether ITG *α*6 contributes to the regulation of NP cells by change of the oxygen content, especially when exposed to high oxygen tension (HOT).

In this study, we found that the levels of ITG *α*6 expression were increased in the NP tissue from IVDD patients and the IVDD rat model with mild degeneration, which were reduced as the degree of degeneration increases in severity. ITG *α*6 expression was also significantly upregulated by HOT through activation of the PI3K/AKT signaling pathway in NP cells. Inhibition of hypoxia-inducible factor-1*α* (HIF-1*α*) by HOT could increase the expression of ITG *α*6, whereas the increase of ITG *α*6 levels downregulated HIF-1*α* expression. Further studies showed that ITG *α*6 was involved in the regulation of NP cell functions, including NP cell apoptosis, Reactive Oxygen Species (ROS) production, and ECM secretion. In addition, silencing of ITG *α*6 accelerated puncture-induced IVDD *in vivo*. Taken together, these data indicate that ITG *α*6 may be a protective factor for the pathogenesis of IVDD.

## 2. Methods and Materials

### 2.1. Reagents and Antibodies

Fetal bovine serum (FBS) was purchased from Biological Industries (Israel). Phosphate-buffered solution (PBS) and Dulbecco's modified Eagle's medium/F12 (DMEM/F12) were purchased from Hyclone (South Logan, UT, USA). Improved Minimal Essential Medium (Opti-MEM), 0.25% trypsin/ethylenediaminetetraacetic acid (EDTA), and type II collagenase were purchased from Gibco (Gaithersburg, MD, USA). TRIzol and Lipofectamine 3000 were purchased from Invitrogen (Carlsbad, CA, USA). The ITG *α*6-specific siRNA duplexes were designed previously and synthesized by RiboBio (Guangzhou, China). PrimeScript RT Master Mix was purchased from Takara Bio Inc. (Shiga, Japan). The JC-1 assay kit, the TUNEL Apoptosis Assay Kit, the Reactive Oxygen Species (ROS) assay kit, the RIPA buffer, the Annexin V-FITC/propidium iodide (PI) staining kit, the PI3K inhibitor (LY294002), and the antibody against HIF-1*α*, Bax, MMP3, and caspase 3 were all purchased from Beyotime Biotechnology (Shanghai, China). The antibodies against Col2*α*1, aggrecan, and *β*-actin were purchased from Abcam (Cambridge, MA, USA). The antibody against ITG *α*6 was purchased from Sabbiotech (College Park, Maryland, USA). The antibody against apoptosis-inducing factor (AIF) was purchased from Cell Signaling Technology (Danvers, MA, USA). The antibody against AKT, p-AKT, and BCL-2 was purchased from ABclonal (Woburn, MA, USA). Chitosan hydrochloride was purchased from Ultrapure Aoxing Bio (Zhejiang, China). Pentasodium tripolyphosphate (TPP) was purchased from Sigma (St. Louis, MO, USA). The HIF-1*α* knockout (HIF-1*α*^−/−^) mouse model was donated by Professor Zhang.

### 2.2. Human IVD Specimen Collection

The degeneration degree of IVDD was assessed according to the Pfirrmann classification system (I-V) [22], which entails the use of T2-weighted magnetic resonance imaging (MRI). To minimize the errors as much as possible, every specimen was assessed by three independent observers. Degenerated specimens were obtained from patients who underwent spinal fusion surgery with low back pain (LBP), and normal disc specimens were obtained from young patients with burst lumbar fracture without IVDD. Finally, twenty-five specimens were collected including 4 healthy discs (19-27 years old, two males and two females) and 21 various degeneration degree discs (22-56 years old, nine males and twelve females). The specimens were divided into four groups: normal (Pfirrmann I, *n* = 4), mild degeneration (Pfirrmann II, *n* = 5), moderate degeneration (Pfirrmann III, *n* = 7), and severe degeneration (Pfirrmann IV-V, *n* = 9).

### 2.3. Establishment of the IVDD Model

The animals used in this study were 3-month-old male Sprague-Dawley (SD) rats, weighing 200–250 g. The annulus needle puncture model of IVDD in the SD rat caudal spine was similar to the previous study [23]. Totally, eighteen rats were randomized as follows: control, 2- and 4-week groups with six rats in each group. After anesthesia with intramuscular injection of 1% pentobarbital sodium at 0.1 mg/kg, a portable X-ray machine was used to check the coccygeal 4-5 disc level (Co4-5), which is the surgical level. A 28-gauge needle was inserted into the center of the disc through the AF, then rotated 360°, and held for 30 s. The rats were euthanized, and the Co4-5 caudal discs were collected. Nine specimens were stored at -80°C for protein extraction for western blotting. The remaining specimens were fixed, decalcified, paraffin-embedded, and sectioned, then were stained with hematoxylin and eosin (HE), Alcian Blue, and immunofluorescence for analysis.

### 2.4. Histology Staining

Both human and rat specimens were fixed with 4% formaldehyde; rat IVD tissues also need to be decalcified with 10% ethylenediaminetetraacetic acid solution for 4 weeks. Then, all specimens were paraffin-embedded for sectioning longitudinally in the sagittal plane at 5 mm intervals. Sections were stained with HE and Alcian Blue to analyze the morphological changes in the discs.

### 2.5. Tissues and Cell Immunofluorescence Staining

Immunofluorescence to assess ITG *α*6, HIF-1*α*, AIF, and Col2*α*1 expression was performed on sections of IVD tissues and fixed cell samples. The fixed cells or sections were blocked with 3% BSA for 1 h, then washed with PBS. Subsequently, samples were incubated overnight with the corresponding antibody mentioned above at 4°C. After three washes with PBS, samples were incubated with a goat anti-rabbit or mouse IgG antibody (1 : 5000, 2 h, room temperature). The samples were then incubated with 0.1 g/ml DAPI for 3 min. After the final round of washes, fluorescence images were captured under a fluorescence microscope (LSM 710, Carl Zeiss, Oberkochen, Germany).

### 2.6. NP Cell Isolation and Culture

NP cells were isolated and cultured as previously described [24]. Briefly, the SD rats were killed with anesthesia overdose. The caudal vertebral spine was totally taken out. Under aseptic conditions, the skin and the musculature attached to the vertebral column were removed to expose the IVD. Then, the annulus fibrosus was incised with bistoury to expose and separate the nucleus pulposus tissues. After cutting the NP tissues into pieces, NP tissues were subsequently digested with 0.25% trypsin with EDTA for 15 min and 0.1% type II collagenase for 2 h at 37°C. NP cells were obtained after filtration of the above residual tissues. Then, the suspension was centrifuged at 1500 rpm for 5 min. The cell pellet was resuspended in DMEM/F12 containing 10% FBS and seeded into a 10 cm plate in a humidified atmosphere of 1% O_2_ and 5% CO_2_, at 37°C. NP cells were adherent to the bottom of the culture dish after 3 days. Cell growth was observed every day under a microscope. The culture medium was replaced by a fresh one for the first time on the 6th day and every 3 days. When confluent after 8-10 days, NP cells were lifted using 0.25% trypsin with EDTA and subcultured to the second generation. And the third generation was used for the following experiments.

### 2.7. Cell Transfection

The Lipofectamine 3000 reagent was used to transiently deliver ITG *α*6 siRNA or negative control (NC) siRNA into NP cells according to the protocol. Briefly, ITG *α*6 or NC siRNA was mixed with the Lipofectamine 3000 reagent and Opti-MEM at room temperature for 15 min. The mixture was added into cells at 90% confluence. After culturing at 37°C and 1% O_2_ for 24 h, total protein was extracted and analyzed by western blot or qRT-PCR to demonstrate the efficiency of transfection.

### 2.8. qRT-PCR

Total RNA was extracted from cell samples using TRIzol according to the manufacturer's instructions, and total RNA was measured spectrophotometrically at 260 nm. mRNA expression was detected by qRT-PCR as previously reported [25]. All data were subsequently normalized to the *β*-actin mRNA level and expressed as relative mRNA expression.

### 2.9. Western Blot

NP tissues separated from the IVD were firstly ground to powder via the grinder. Then, NP cells or NP tissues were lysed in the RIPA buffer containing the protease and phosphatase inhibitor for 30 min at 4°C. The supernatant was carefully collected in order to acquire total protein. Protein concentration was measured by the BCA protein assay kit. The samples were electrophoresed on SDS-PAGE and transferred to PVDF membranes. The membranes were blocked with 5% nonfat milk for 30 min to block nonspecific antigens followed by corresponding primary antibodies and incubated overnight at 4°C. After washing in TBST 3 times for 10 min, the membrane was incubated with a secondary anti-IgG-HRP antibody at room temperature for 2 h. Immunolabeling was detected using an enhanced chemiluminescence reagent.

### 2.10. Flow Cytometric Analysis for the Apoptosis Percentage and ROS Production Level

The apoptosis percentage of NP cells was analyzed using an Annexin V-FITC/propidium iodide (PI) staining kit, and the ROS production level was analyzed using a Reactive Oxygen Species assay kit; both experiments were performed following the manufacturer's protocol. Briefly, after being digested with 0.25% trypsin and centrifuged (1500 rpm for 5 min), NP cells were obtained and then incubated with Annexin V-FITC for 10 min and then PI for 10 min for apoptosis percentage analysis or incubated with DCFH-DA for 20 min for ROS production level detection. Stained cells were quantified by flow cytometry.

### 2.11. TUNEL

The cells treated as indicated were fixed with 4% paraform phosphate-buffered saline, rinsed with PBS, and then permeabilized by 0.1% Triton X-100 for FITC end-labeling the fragmented DNA of the apoptotic NP cells using the TUNEL cell apoptosis detection kit. The FITC-labeled TUNEL-positive cells were imaged under a fluorescence microscope by using 488 nm excitation and 530 nm emission.

### 2.12. Mitochondrial Membrane Potential Assay with JC-1

The mitochondrial membrane potential was detected with a Mitochondrial Membrane Potential Assay Kit. In brief, the NP cells were seeded in 6-well plates. Following different treatments, the media were removed and then were stained with JC-1 for 30 min. Subsequently, the cells were washed with a serum-free medium three times, and the fluorescence intensity (excitation 585 nm, emission 590 nm) was detected by a fluorescence microplate reader.

### 2.13. Detection of the ROS Production Level

ROS was estimated by a Reactive Oxygen Species assay kit according to the manufacturer's instructions. The NP cells were seeded in 6-well plates. Following different treatments, the media were removed, and the cells were loaded with DCFH-DA (10 *μ*M) at 37°C for 20 min. Subsequently, the cells were washed with a serum-free medium three times, and the fluorescence intensity (excitation 488 nm, emission 525 nm) was detected by a fluorescence microplate reader.

### 2.14. Chitosan Nanoparticles for siRNA Delivery Preparation

Nanoparticles were produced based on modified ionic gelation of TPP with chitosan as previously described [26]. Briefly, chitosan hydrochloride was dissolved with distilled water to get chitosan solution (10 ml, 2 mg/ml); then, the solution was centrifugated at 13,000 × g for 10 min. The supernatants were discarded, and chitosan nanoparticles were resuspended in filtered distilled water. Do the same to get TPP aqueous solution (10 ml, 0.84 mg/ml). Nanoparticles were spontaneously obtained upon the addition of 0.4 ml of TPP aqueous solution (0.84 mg/ml) to 1 ml of chitosan solution (2 mg/ml, at chitosan to TPP weight ratio of 6 : 1) under constant magnetic stirring at room temperature. For the association of siRNA with the chitosan-TPP nanoparticles (chitosan-TPP-siRNA), 20 *μ*l of siRNA (1 *μ*g/*μ*l) in RNase-free water was added to the TPP solution (0.4 ml, 0.84 mg/ml) before adding this to the chitosan solution (1 ml, 2 mg/ml). The particles were then incubated at room temperature for 30 min before further use.

### 2.15. Injection of Chitosan-TPP-siRNA Nanoparticles in the IVDD Rat Model

All surgical procedures were similar to the protocol used in the establishment of the IVDD rat tail model as described before. At last, 4 *μ*l of chitosan-TPP-siRNA nanoparticles or PBS was injected into the surgery level. Two weeks after surgery, specimens were obtained.

### 2.16. Statistical Analysis

Data were collected from three or more independent experiments and expressed as mean ± SD. A two-sided Student's *t*-test was used to analyze the difference for experiments with two subgroups. One-way analysis of variance (ANOVA) was performed to show the difference for experiments with more than two subgroups.

## 3. Results

### 3.1. Upregulation of ITG *α*6 Expression by HOT in NP Cells through Activation of the PI3K/AKT Signaling Pathway *In Vitro*

**Figure 1:**
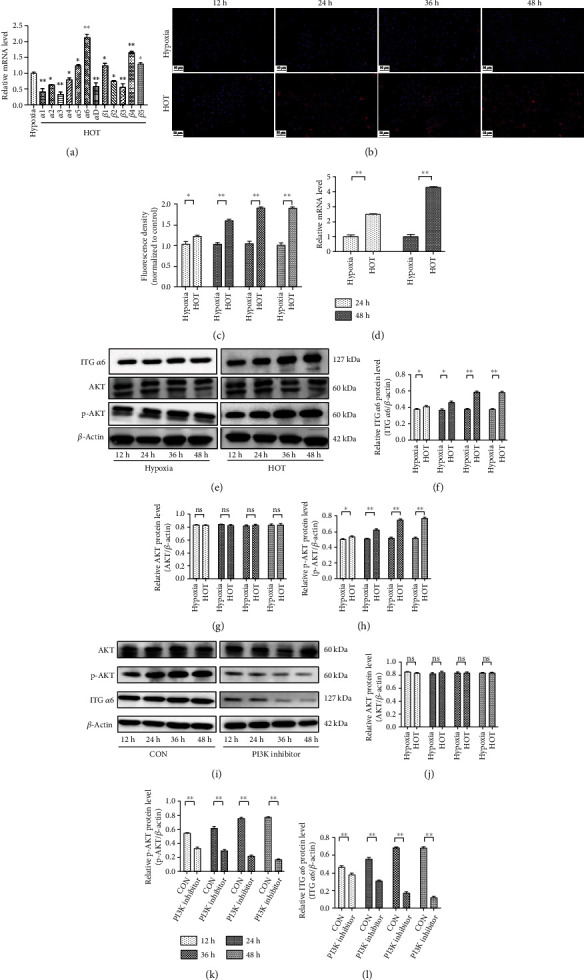
Upregulation of ITG *α*6 expression by HOT in NP cells through activation of the PI3K/AKT signaling pathway *in vitro*. (a) qRT-PCR analysis of twelve known subunits of the integrins in NP cells exposed to HOT (21%, O_2_) for 24 h. *n* = 4. ^∗^*P* < 0.05, ^∗∗^*P* < 0.01. (b) Expression of ITG *α*6 was observed by immunofluorescence staining in NP cells exposed to HOT (21%, O_2_) for different time courses. *n* = 4. (c) Quantitative analysis of positive staining. The intensity of the immunofluorescence of ITG *α*6 was markedly increased in NP cells exposed to HOT (21%, O_2_) from 12 h to 48 h. *n* = 4. ^∗^*P* < 0.05, ^∗∗^*P* < 0.01. (d) qRT-PCR analysis of ITG *α*6 mRNA expression in NP cells exposed to HOT for 24 h and 48 h. *n* = 4. ^∗∗^*P* < 0.01. (e) Western blot analysis of ITG *α*6, AKT, p-AKT, and *β*-actin expression in NP cells exposed to HOT (21%, O_2_) for different time courses. *n* = 4. (f–h) The results of western blot analysis are expressed as percentages of positive mean values ± SD. *n* = 3. ^∗∗^*P* < 0.01, ^∗^*P* < 0.05. (i) Western blot analysis of ITG *α*6, AKT, p-AKT, and *β*-actin expression in NP cells treated with (LY294002, 20 *μ*M) under HOT (21%, O_2_). *n* = 4. (j–l) The results of western blot analysis are expressed as percentages of positive mean values ± SD. *n* = 3. ^∗∗^*P* < 0.01, ^∗^*P* < 0.05.

A panel of qRT-PCR assays was used to measure twelve known subunits of the ITG in NP cells exposed to HOT (21%, O_2_) for 24 h. All of these subunits were differentially expressed in NP cells in response to HOT, in which ITG *α*5, ITG *α*6, ITG *β*1, ITG *β*4, and ITG *β*5 were upregulated, but ITG *α*1, ITG *α*2, ITG *α*3, ITG *α*4, ITG *α*D, ITG *β*2, and ITG *β*3 were downregulated (Figure 1(a)). Among these differentially expressed members, ITG *α*6 was found to be upregulated by more than twofold. Further studies showed that ITG *α*6 expression was increased by HOT in a time-dependent manner in NP cells, as evidenced by the immunofluorescence staining (Figures 1(b) and 1(c)). qRT-PCR and western blot results also showed that HOT significantly increased ITG *α*6 expression in NP cells in a time-dependent manner (Figures 1(d)–1(f)). Furthermore, we found that the expression of p-AKT was increased in NP cells exposed to HOT from 12 h to 48 h (Figures 1(e)–1(h)). Blocking the PI3K/AKT signaling pathway with the PI3K inhibitor could obviously alleviate the upregulation of ITG *α*6 expression by HOT (Figures 1(i)–1(l)), indicating that HOT upregulated the expression of ITG *α*6 in NP cells through activation of the PI3K/AKT signaling pathway.

### 3.2. The Expression of ITG *α*6 Is Increased in the NP Tissue from Patients with Mild Degeneration of IVDD

**Figure 2:**
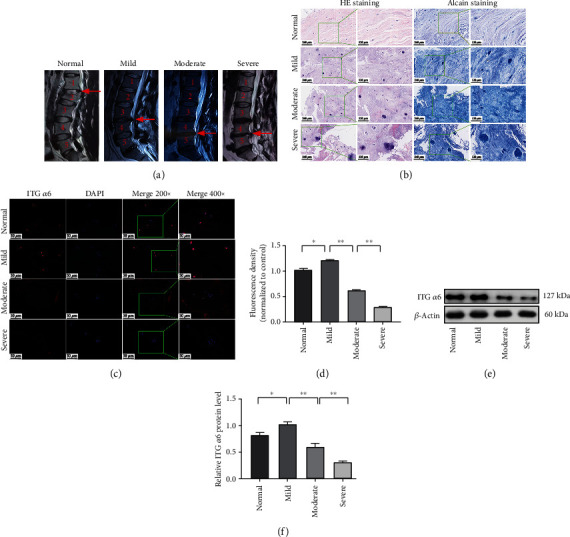
The expression of ITG *α*6 is increased in the NP tissue from patients with mild degeneration of IVDD. (a) Representative T2-weighted MRI of human NP tissues graded by the Pfirrmann system: normal (Pfirrmann I), mild degeneration (Pfirrmann II), moderate degeneration (Pfirrmann III), and severe degeneration (Pfirrmann IV-V). (b) HE staining and Alcian Blue staining of human NP tissues with different degrees of IVDD. *n* = 3. (c) Immunofluorescence staining of ITG *α*6 in human NP tissue with different degrees of IVDD. (d) Quantitative analysis of positive immunofluorescence staining. *n* = 3. ^∗∗^*P* < 0.01, ^∗^*P* < 0.05. (e) Western blot analysis of ITG *α*6 in human NP tissue with different degrees of IVDD. (f) The results of western blot analysis are expressed as percentages of positive mean values ± SD. *n* = 3. ^∗∗^*P* < 0.01, ^∗^*P* < 0.05.

IVDD based on MRI data was graded by using the Pfirrmann system. A total of 25 disc NP samples were divided into four groups: normal (Pfirrmann I, *n* = 4), mild degeneration (Pfirrmann II, *n* = 5), moderate degeneration (Pfirrmann III, *n* = 7), and severe degeneration (Pfirrmann IV-V, *n* = 9) (Figure 2(a)). HE staining and Alcian Blue staining of NP tissue sections were used to evaluate the pathologic change of the IVD tissue. The results showed that the gelatine NP gradually disappeared and fibrocartilage was formed on the surface of cartilage in the IVDD group compared to the normal group, which appeared in an IVDD degree-dependent manner (Figure 2(b)). Subsequently, immunofluorescence staining was applied to investigate the localization and expression of ITG *α*6 in NP tissue from patients with different degrees of IVDD. The results showed that the intensity of the immunofluorescence of ITG *α*6 was increased in the NP tissue from IVDD patients with mild degeneration. However, ITG *α*6 expression was decreased in human moderate and severe degenerated NP tissue (Figures 2(c) and 2(d)). Similar results were also examined by western blot (Figures 2(e) and 2(f)). These results indicated that the level of ITG *α*6 was increased in the early stage of disc degeneration, whereas it was reduced in the middle and late stages of disc degeneration.

### 3.3. The Expression of ITG *α*6 Is Increased in the NP Tissue from the Rat Model with Mild Degeneration of IVDD

**Figure 3:**
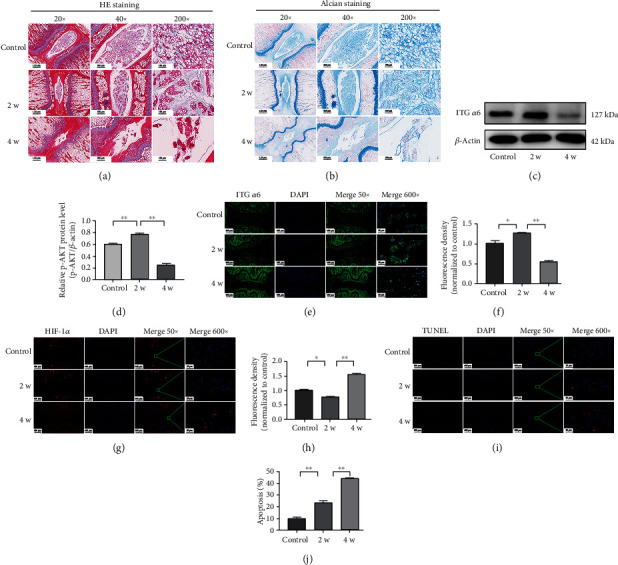
The expression of ITG *α*6 is increased in the NP tissue from the rat model with mild degeneration of IVDD. (a, b) HE staining and Alcian Blue staining of NP tissues in the rat model at 2 and 4 weeks after annulus needle puncture. (c) Western blot analysis of ITG *α*6 and *β*-actin expression of NP tissues in the rat model. (d) The results of western blot analysis are expressed as percentages of positive mean values ± SD. *n* = 4. ^∗^*P* < 0.05, ^∗∗^*P* < 0.01. (e) Immunofluorescence staining of ITG *α*6 in NP tissue in the rat model. (f) Quantitative positive immunofluorescence staining analysis of ITG *α*6. *n* = 4. ^∗^*P* < 0.05, ^∗∗^*P* < 0.01. (g) Immunofluorescence staining of HIF-1*α* in NP tissue in the rat model. (h) Quantitative positive immunofluorescence staining analysis of HIF-1*α*. *n* = 4. ^∗^*P* < 0.05, ^∗∗^*P* < 0.01. (i) TUNEL staining of NP tissues to examine the apoptosis in the rat model. (j) Quantitative positive staining analysis of TUNEL staining. *n* = 4. ^∗^*P* < 0.05, ^∗∗^*P* < 0.01.

To further examine the expression of ITG *α*6 in IVDD, an IVDD rat model was established by annulus needle puncture. Two and four weeks after puncture, the gelatine NP gradually disappeared and fibrocartilage was formed on the surface of cartilage, accompanied by the disappearance of the border between NP and AF, as evidenced by HE staining and Alcian Blue staining (Figures 3(a) and 3(b)). These results indicated that the IVDD rat model was successfully made in this study. Next, we tested the expression of ITG *α*6 in NP tissue from the IVDD rat model at two and four weeks after puncture. The results of western blot showed that the level of ITG *α*6 was significantly upregulated in NP tissue from the IVDD rat model at two weeks after puncture, which was decreased in the IVDD rat model at four weeks after puncture (Figures 3(c) and 3(d)); similar results were tested by the immunofluorescence staining (Figures 3(e) and 3(f)). In addition, we tested the expression of HIF-1*α* in NP tissue from the IVDD rat model. We found that HIF-1*α* expression was decreased in the IVDD rat model at two weeks after puncture, which was increased at four weeks after puncture (Figures 3(g) and 3(h)). In addition, TUNEL staining was applied to examine the apoptosis in NP tissue from the IVDD rat model. We found that the apoptotic rate of the NP cells was significantly increased in NP tissue from the IVDD rat model at two weeks after puncture, which was further increased after four weeks (Figures 3(i) and 3(j)).

### 3.4. Activation of the PI3K/AKT/ITG *α*6 Pathway Protects NP Cells against HOT-Inhibited ECM Protein Synthesis

**Figure 4:**
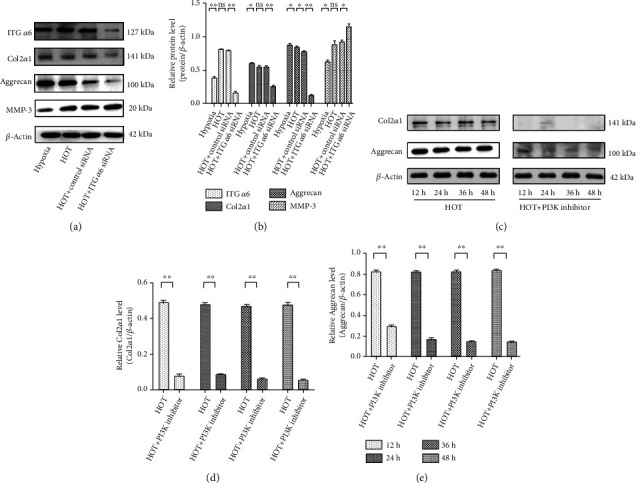
Activation of the PI3K/AKT/ITG *α*6 pathway protects NP cells against HOT-inhibited ECM protein synthesis. (a) Western blot analysis of ITG *α*6, Col2*α*1, aggrecan, MMP3, and *β*-actin expression in NP cells treated with HOT and siRNA-ITG *α*6. (b) The results of western blot analysis are expressed as percentages of positive mean values ± SD. *n* = 3. ^∗^*P* < 0.05, ^∗∗^*P* < 0.01. (c) Western blot analysis of Col2*α*1, aggrecan, and *β*-actin expression in NP cells treated with HOT and the PI3K inhibitor (LY294002, 20 *μ*M) for 24 h and 48 h. (d, e) The results of western blot analysis are expressed as percentages of positive mean values ± SD. *n* = 3. ^∗^*P* < 0.05, ^∗∗^*P* < 0.01.

To investigate the role of ITG *α*6 in the secretion of ECM, the level of ITG *α*6 was decreased with siRNA-ITG *α*6. The western blot results showed that ITG *α*6 expression was obviously decreased in NP cells transfected with siRNA-ITG *α*6 compared to NP cells transfected with siRNA-NC, indicating that the deletion of ITG *α*6 was effective (Figures 4(a) and 4(b)). The ECM consists primarily of aggregating proteoglycans, Col2*α*1 and MMP3. The western blot results showed that HOT decreased the expression of aggrecan and Col2*α*1, whereas they were further decreased by silencing of ITG *α*6, while expression of MMP3 was totally opposite, suggesting that ITG *α*6 could alleviate the inhibitory effect of HOT on ECM secretion (Figures 4(a) and 4(b)). Furthermore, the decrease of aggrecan and Col2*α*1 expression was observed in NP cells exposed to HOT from 12 h to 48 h, which could be aggravated by the PI3K inhibitor (Figures 4(c)–4(e)), suggesting that activation of the PI3K/AKT/ITG *α*6 pathway had a protective effect on NP cells against HOT-inhibited ECM protein synthesis.

### 3.5. ITG *α*6 Protects NP Cells from Apoptosis Induced by HOT

**Figure 5:**
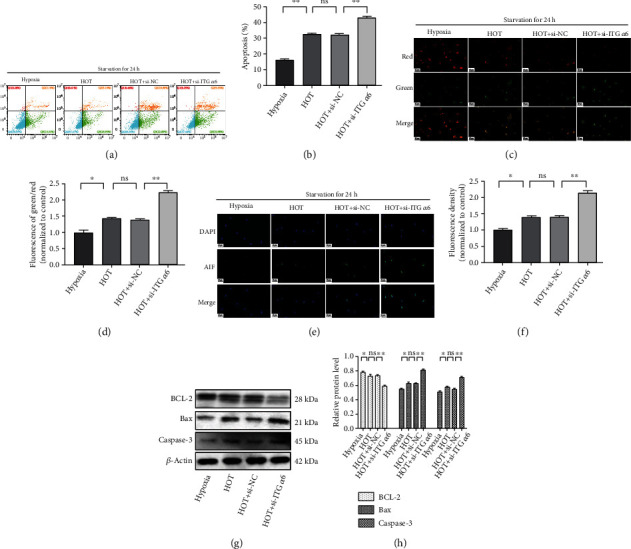
ITG *α*6 protects NP cells from apoptosis induced by HOT. (a, b) NP cells treated with HOT and siRNA-ITG *α*6 under serum-free conditions were stained with Annexin V and PI and detected by flow cytometric analysis. The apoptosis percentage was calculated according to the result of flow cytometric analysis. Data were presented as the mean ± SD. *n* = 3. ^∗^*P* < 0.05, ^∗∗^*P* < 0.01. (c, d) NP cells treated with HOT and siRNA-ITG *α*6 under serum-free conditions were stained with JC-1. Quantitative analysis of the green (high ΔΨm) and red (low ΔΨm) staining. The green (high ΔΨm) to red (low ΔΨm) ratio markedly increased in NP cells treated with siRNA-ITG *α*6 and HOT. *n* = 3. ^∗^*P* < 0.05, ^∗∗^*P* < 0.01. (e) Immunofluorescence staining of AIF in NP cells treated with HOT and siRNA-ITG *α*6 under serum-free conditions. (f) Quantitative analysis of positive immunofluorescence staining. *n* = 3. ^∗^*P* < 0.05, ^∗∗^*P* < 0.01. (g) Western blot analysis of BCL-2, Bax, caspase 3, and *β*-actin expression in NP cells treated with HOT and siRNA-ITG *α*6. (h) The results of western blot analysis are expressed as percentages of positive mean values ± SD. *n* = 3. ^∗^*P* < 0.05, ^∗∗^*P* < 0.01.

To investigate whether ITG *α*6 was involved in HOT-induced NP cell apoptosis, flow cytometric analysis was firstly applied to monitor cell apoptosis. A population of apoptotic NP cells presented phosphatidylserine (PS) externalization as evidenced by a significant increase in Annexin-FITC fluorescence, as well as decreased plasma membrane integrity as determined by a modest increase in propidium iodide fluorescence. We found that HOT obviously promoted serum deprivation-induced NP cell apoptosis, whereas silencing of ITG *α*6 further aggravated HOT-induced NP cell apoptosis (Figures 5(a) and 5(b)). Subsequently, the quantitative analysis of JC-1-stained cells revealed a significant increase in the green (high ΔΨm) to red (low ΔΨm) ratio in HOT-treated cells when compared with hypoxia cells. Silencing of ITG *α*6 could further increase the green (high ΔΨm) to red (low ΔΨm) ratio in HOT-treated cells, suggesting that ITG *α*6 could protect NP cells from HOT-induced apoptosis (Figures 5(c) and 5(d)). Apoptosis-inducing factor (AIF) was involved in mitochondrial respiration and caspase-independent apoptosis. The results of immunofluorescence staining showed that the nuclear accumulation of AIF in NP cells was markedly increased by HOT. Silencing of ITG *α*6 enhanced HOT-induced nuclear accumulation of AIF in NP cells (Figures 5(e) and 5(f)). The effects of silencing of ITG *α*6 on cell apoptosis-related protein (BCL-2, Bax, and caspase 3) expression were observed by western blot in NP cells exposed to HOT. HOT increased the expressions of Bax and caspase 3 and decreased the expression of BCL-2, which were further aggravated in NP cells with silenced expression of ITG *α*6, indicating that silencing of ITG *α*6 further promoted HOT-induced NP cell apoptosis (Figures 5(g) and 5(h)). Taken together, these results indicated that ITG *α*6 protected NP cells from apoptosis induced by HOT.

### 3.6. ITG *α*6 Protects against High Oxygen Tension-Induced ROS Production in NP Cells

**Figure 6:**
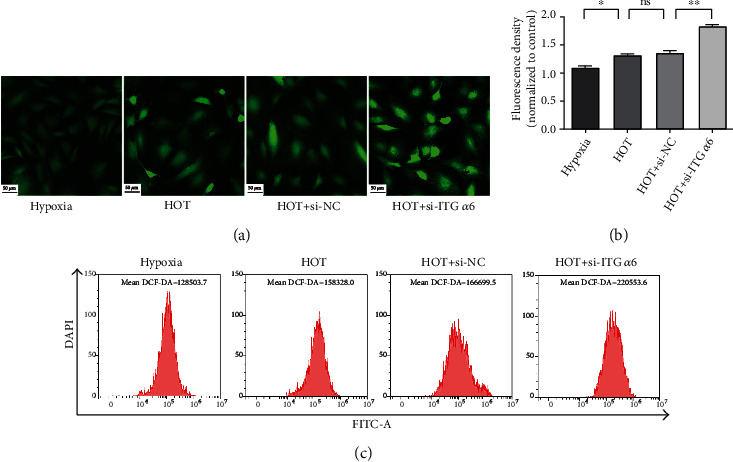
ITG *α*6 protects against high oxygen tension-induced ROS production in NP cells. (a) NP cells treated with HOT and siRNA-ITG *α*6 were stained with DCF-DA and detected by a fluorescence microscope. (b) Data were shown as mean ± SD. *n* = 3. ^∗^*P* < 0.05, ^∗∗^*P* < 0.01. (c) NP cells treated with HOT and siRNA-ITG *α*6 were stained with DCF-DA and detected by flow cytometric analysis.

To investigate the effect of ITG *α*6 on HOT-induced ROS production in NP cells, 2′,7′-dichlorodihydrofluorescein diacetate (DCF-DA) staining was used to test the level of ROS. The fluorescence intensity of DCF-DA was observed with a fluorescence microscope. The fluorescence intensity of DCF-DA was increased in NP cells exposed to HOT for 24 h. Silencing of ITG *α*6 could further enhance the promoting effect of HOT on the production of ROS in NP cells (Figures 6(a) and 6(b)). We also applied flow cytometry to measure the fluorescence intensity of DCF-DA. The results showed that the peak fluorescence intensity of DCF-DA on flow cytometry was increased in NP cells exposed to HOT for 24 h. Induction of ROS by HOT was further increased in ITG *α*6 silenced NP cells, indicating that ITG *α*6 could protect against HOT-induced ROS production in NP cells (Figure 6(c)).

### 3.7. The Existence of an Interaction and Feedback Regulation between HIF-1*α* and ITG *α*6 in NP Cells Exposed to HOT

**Figure 7:**
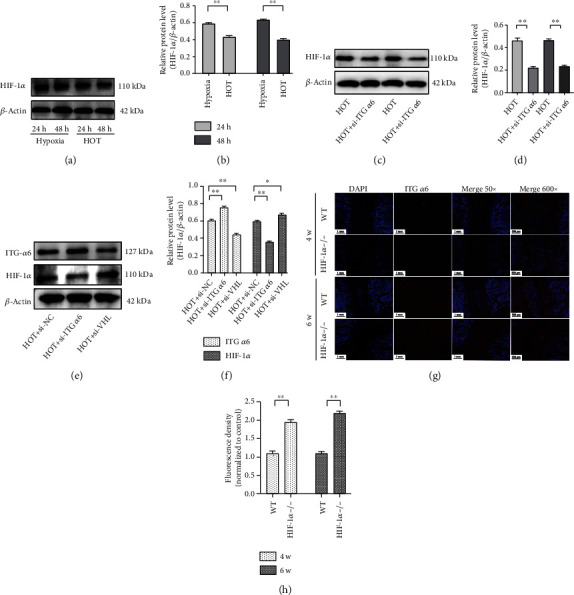
The existence of an interaction and feedback regulation between HIF-1*α* and ITG *α*6 in NP cells exposed to HOT. (a) Western blot analysis of HIF-1*α* and *β*-actin expression in NP cells treated with HOT for 24 h and 48 h. (b) The results of western blot analysis are expressed as percentages of positive mean values ± SD. *n* = 3. ^∗^*P* < 0.05, ^∗∗^*P* < 0.01. (c) Western blot analysis of HIF-1*α* and *β*-actin expression in NP cells treated with siRNA-ITG *α*6 and HOT. (d) The results of western blot analysis are expressed as percentages of positive mean values ± SD. *n* = 3. ^∗^*P* < 0.05, ^∗∗^*P* < 0.01. (e) Western blot analysis of HIF-1*α*, ITG *α*6, and *β*-actin expression in NP cells treated with siRNA-HIF-1*α* or VHL and HOT. (f) The results of western blot analysis are expressed as percentages of positive mean values ± SD. *n* = 3. ^∗^*P* < 0.05, ^∗∗^*P* < 0.01. (g) Immunofluorescence staining of ITG *α*6 in the knockout (HIF-1*α*^−/−^) mouse model and the WT mouse. (h) Quantitative analysis of positive staining. The intensity of the immunofluorescence of ITG *α*6 was markedly increased in the HIF-1*α* knockout (HIF-1*α*^−/−^) mouse model. *n* = 3. ^∗^*P* < 0.05, ^∗∗^*P* < 0.01.

The expression of HIF-1*α* was significantly decreased in NP cells exposed to HOT compared to cells cultured in hypoxia (Figures 7(a) and 7(b)). Silencing of ITG *α*6 obviously enhanced the inhibitory effect of HOT on HIF-1*α* expression, suggesting that ITG *α*6 could positively regulate the expression of HIF-1*α* (Figures 7(c) and 7(d)). However, we found that silencing of HIF-1*α* could obviously upregulate ITG *α*6 expression, indicating that HIF-1*α* could negatively regulate the expression of ITG *α*6 (Figures 7(e) and 7(f)). Similar results were also examined in the HIF-1*α* knockout (HIF-1*α*^−/−^) mouse model. The results of immunofluorescence staining showed that a strong red fluorescence signal of ITG *α*6 arose from the NP tissue of the HIF-1*α*^−/−^ mouse compared with the WT mouse (Figures 7(g) and 7(h)). Based on the above results, we concluded that the increase of ITG *α*6 expression by HOT contributed to maintaining NP tissue homeostasis through the interaction with HIF-1*α*.

### 3.8. Silencing of ITG *α*6 Accelerates Puncture-Induced IVDD *In Vivo*

**Figure 8:**
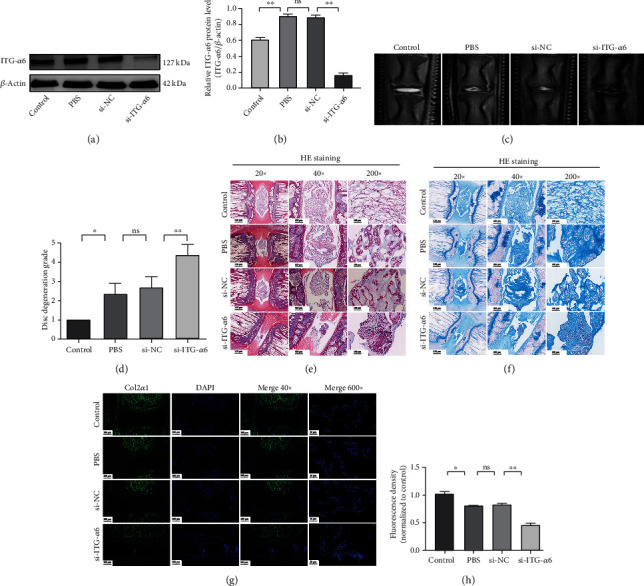
Silencing of ITG *α*6 accelerates puncture-induced IVDD *in vivo*. (a) Western blot analysis of ITG *α*6 and *β*-actin expression of NP tissues in the rat model treated with siRNA-ITG *α*6-CH. (b) The results of western blot analysis are expressed as percentages of positive mean values ± SD. *n* = 3. ^∗^*P* < 0.05, ^∗∗^*P* < 0.01. siRNA-ITG *α*6-CH injected into IVD significantly decreased the expression of ITG *α*6 *in vivo*. (c) MRI was used to investigate disc changes and then the Pfirrmann score. (d) The data of the Pfirrmann score were expressed as percentages of positive mean values ± SD. *n* = 3. ^∗^*P* < 0.05, ^∗∗^*P* < 0.01. (e, f) HE and Alcian Blue staining showed the severity of IVDD changes at 2 weeks after puncture compared with the control group. (g) Immunofluorescence staining of Col2*α*1 at 2 weeks after puncture compared with the control group. (h) Quantitative analysis of positive staining. The intensity of the immunofluorescence of Col2*α*1was markedly decreased in the rat model treated with siRNA-ITG *α*6-CH. *n* = 3. ^∗^*P* < 0.05, ^∗∗^*P* < 0.01.

To further determine the effects of ITG *α*6 on IVDD *in vivo*, siRNA-ITG *α*6 nanoparticles (siRNA-ITG *α*6-CH) were injected into the intervertebral disc to decrease ITG *α*6 expression *in vivo*. The inhibitory effects of siRNA-ITG *α*6-CH in NP tissue were examined by western blot. The results showed that the expression of ITG *α*6 was decreased in the NP tissues from the rat injected with siRNA-ITG *α*6-CH compared with the rat injected with siRNA-NC-CH (Figures 8(a) and 8(b)). To observe the effect of decreasing ITG *α*6 *in vivo* in the rat puncture model, MRI was firstly used to determine disc changes and then the Pfirrmann score. MRI results showed that the Pfirrmann score of siRNA-ITG *α*6-CH rats was significantly higher than that of siRNA-NC-CH rats at 2 weeks after puncture (Figures 8(c) and 8(d)), indicative of more severe IVDD. HE and Alcian Blue staining showed severe IVDD changes at 2 weeks after puncture in contrast to the control group. The siRNA-ITG *α*6-CH group displayed significantly decreased NP content compared with the siRNA-NC-CH group at 2 weeks after puncture (Figures 8(e) and 8(f)). Col2*α*1 immunofluorescence also showed significantly decreased Col2*α*1 content in the siRNA-ITG *α*6-CH group (Figures 8(g) and 8(h)). Taken together, these results demonstrated that ITG *α*6 might be a protective factor for IVDD.

## 4. Discussion

Low back pain due to IVDD affects many individuals, especially in the elderly population [2]. It often leads to a lower quality of life, which meanwhile results in direct and indirect annualized costs amounting to $50 billion in the United States [1]. Identifying the mechanisms of IVDD and finding new therapeutic targets is imperative. The present study demonstrated for the first time that the expression of ITG *α*6 was significantly increased in IVDD at an early degeneration stage. The increase of oxygen content of NP tissue was a critical factor for upregulation of ITG *α*6 in IVDD. Further studies found that ITG *α*6 could protect NP cells against HOT-induced apoptosis and ROS and protect NP cells from HOT-inhibited ECM protein synthesis. Upregulation of ITG *α*6 expression by HOT contributed to maintaining NP tissue homeostasis through the interaction with HIF-1*α*. Furthermore, silencing of ITG *α*6 *in vivo* could obviously accelerate puncture-induced IVDD. Taken together, these results revealed that the increase of ITG *α*6 expression by HOT in NP cells might be a protective factor in IVD degeneration as well as restore NP cell function.

The latest study showed that 5% and 21% oxygen concentration led to a decrease in anabolic gene expression of NPs while 1% led to an increase, indicating that hypoxia was the most suitable and biological environment to maintain the NP phenotype and normoxia (21%, O_2_, defined as HOT in this study) was a pathological environment to NP cells [27]. The increase of oxygen tension caused by abnormal neovascularization in discs is strongly related to the establishment and progression of IDD [28]. Currently, most studies focus on the protective effect of hypoxia on NPs [29–31]. However, very few studies were carried out to investigate the exquisite adaptation to oxygen stress of NP cells. In the present study, we explored which might be the most affected genes in the ITG family by “adverse” oxygen conditions and might provide a potential target for treating IVDD. In addition, our results showed that HOT could increase ROS production and inhibit ECM protein synthesis. These results were consistent with previous studies, indicating that the cell model of HOT used in this study was successful and the results from these cell models were reasonable and reliable.

Many cytokines and chemokines have been confirmed to regulate cell metabolism by upregulating the expression levels of integrins [13, 35-36]. Jovanovi et al. found that interleukin-8 stimulated trophoblast cell migration and invasion by increasing levels of MMP2, MMP9, ITG *α*5, and ITG *β*1 [32]. Li et al. confirmed that CCL17 induced trophoblast migration and invasion by regulating matrix metalloproteinase and integrin expression in the human first-trimester placenta [33]. However, no studies reported that the change of the oxygen gradient could serve as a stimulator to regulate integrin expression and is involved in cell metabolism and growth. In the present study, we demonstrated for the first time that HOT increased the expression of ITG *α*6 in NP cells of IVDD. We further found that HOT upregulated ITG *α*6 expression by activating the PI3K/AKT signaling pathway, which played a key role in IVDD. In addition, we found that the level of ITG *α*6 was increased in the early stage of IVDD, whereas it was reduced in the middle and late stages of disc degeneration *in vivo*. It is likely that the upregulation of ITG *α*6 is a stress response of NP cells exposed to HOT in the early stage of degeneration. With the progress of IVDD, NP cells are subject to more severe microenvironmental stresses including starvation and oxygen stress due to the endplate calcification and metabolic waste accumulation [34], resulting in severe destruction of NP tissue, which may be one of the reasons for the low ITG *α*6 expression in the middle and late stages of disc degeneration.

NP cells express HIF-1*α*, a key transcription factor that regulates anaerobic metabolism [35]. Unlike other skeletal cells, NP cells stably express HIF-1*α* in normoxia while slightly increase induction in HIF-1*α* protein levels in hypoxia [36]. Brooks et al. reported that the expression of integrin *α*6 was positively regulated by HIF-1*α* in breast stem cells [37]. However, inconsistent with Brooks et al.'s report from breast stem cells, ITG *α*6 was found to have an interaction and feedback regulation effect on HIF-1*α* in NP cells exposed to HOT. Silencing ITG *α*6 resulted in a decrease in the expression of HIF-1*α*. In reverse, silencing the expression of HIF-1*α* led to elevated expression of ITG *α*6. Therefore, this might explain why HIF-1*α* was expressed constantly in normoxia in NP cells. Previous studies demonstrated that HIF-1*α* played an important role in NP cell metabolic regulation [36]. It promoted the glycolytic pathway by increasing the expression of numerous glycolytic enzymes, which is important for NP cell survival [38, 39]. It is possible that increased expression of ITG *α*6 in NP cells in response to HOT contributes to maintaining NP tissue homeostasis and NP cell survival through the interaction with HIF-1*α*.

The PI3K/AKT signaling pathway, which regulates a wide range of cellular functions, is involved in the resistance response to hypoxia [40]. In this study, integrin *α*6 was also proved to be a downstream protein of the PI3K/AKT signaling pathway, and it is in the opposite way as HIF-1*α*, influenced by the oxygen gradient. HOT activated the PI3K/AKT pathway, and silencing the PI3K/AKT signaling pathway would reduce the level of ITG *α*6. This indicated that the PI3K/AKT pathway was involved in the regulation of the expression of ITG *α*6. HIF-1*α* is also regulated by the PI3K/AKT pathway; however, expression of HIF-1*α* is elevated constantly in hypoxia, and ITG *α*6 is elevated at the early phase of HOT. The knockout of HIF-1*α* resulted in increased integrin *α*6, and this indicates both integrin *α*6 and HIF-1*α* regulated by the PI3K/AKT pathway have a mutual effect on adjustment of NP cell metabolism, which protects the cell from apoptosis when the hypoxia environment is lost.

There are several limitations of the present study. Firstly, although our results support that ITG *α*6 could ameliorate IVDD by regulating ROS production and apoptosis, however, the particular downstream regulation mechanism remains unclear. Secondly, the underlying interaction between HIF-1*α* and ITG *α*6 needs further elucidation. Lastly, the role of ITG *α*6 in IVDD requires a more detailed *in vivo* study.

In conclusion, the mechanisms of IVDD remain unknown and require further illumination. In the present study, we confirmed that the expression of ITG *α*6 was increased in the early stage of disc degeneration. The destruction of the hypoxia environment in NP tissue is a leading cause of increased expression of ITG *α*6. Furthermore, we demonstrated that ITG *α*6 could protect NP cells against HOT-induced apoptosis and oxidative stress and protect NP cells from HOT-inhibited ECM protein synthesis. Silencing of ITG *α*6 *in vivo* obviously accelerates puncture-induced IVDD. Taken together, our study demonstrates for the first time that the ITG *α*6 is a crucial protective factor for IVDD. These findings may provide new insights into the development of pharmacologic and physical therapies that can modify the course of IVDD.

## Data Availability

The authors provide a detailed description of methods and original data upon request.
